# Geographic Disparities in Oral Cancer Survival From 10 Population-Based Cancer Registries in India

**DOI:** 10.1001/jamanetworkopen.2025.3910

**Published:** 2025-04-08

**Authors:** Krishnan Sathishkumar, Jayasankar Sankarapillai, Stephen Santhappan, Aleyamma Mathew, Rekha A. Nair, Nitin Gangane, Sushma Khuraijam, Debanjana Barman, Shashank Pandya, Gautam Majumdar, Vinay Deshmane, Eric Zomawia, Ashok T. Sherpa, Preethi George, Swapna Maliye, Tashnin Rahman, Anand Shah, Shravani Koyande, Lalawmpuii Pachuau, Priya D. Pradhan, Shalin Lily Giboy, Prashant Mathur

**Affiliations:** 1 National Centre for Disease Informatics and Research, Indian Council of Medical Research, Bengaluru, India; 2 Regional Cancer Centre, Thiruvananthapuram, India; 3 Mahatma Gandhi Institute of Medical Sciences, Sevagram, India; 4 Regional Institute of Medical Sciences, Imphal, India; 5 Dr B. Borooah Cancer Institute, Guwahati, India; 6 The Gujarat Cancer and Research Institute, Ahmedabad, India; 7 Regional Cancer Centre, Agartala, India; 8 Indian Cancer Society, Mumbai, India; 9 National Health Mission, Health and Family Welfare, Government of Mizoram, Aizawl, India; 10 New STNM (Sir Thutob Namgyal Memorial) Hospital, Gangtok, India

## Abstract

**Importance:**

India accounts for more than one-third of oral cancer (OC) cases globally. Cancer survival measures the effectiveness of the health care system’s cancer control efforts and the proportion of people who survive during a specified time.

**Objective:**

To estimate the 5-year survival rates among patients with OC diagnosed in India and assess the association of survival with age, place of residence, histologic type, and clinical extent of OC.

**Design, Setting, and Participants:**

This retrospective cohort study evaluated survival of patients diagnosed with primary OC (*International Classification of Diseases for Oncology, Third Revision,* codes C01-C06) between January 1, 2012, and December 31, 2015, and followed up until June 30, 2021. Patients were included from 10 population-based cancer registries (PBCRs). Data were analyzed between March 15 and August 20, 2024.

**Exposures:**

Demographic factors (patient age, sex, and place of residence) and disease factors (clinical extent of disease and histologic type).

**Main Outcomes and Measures:**

The main outcome was 5-year age-standardized relative survival (ASRS) for OC among male and female patients. A multivariable Cox proportional hazards model estimated hazard ratio (HR) and 95% CI, adjusting for covariates.

**Results:**

Data from the 10 PBCRs encompassed 14 059 patients of all ages (median age, 55 [IQR, 45-65] years; 10 380 male [73.8%]) diagnosed with OC. The overall 5-year ASRS rate for OC was 37.2% (range, 20.9%-58.4%). The estimated ASRS rates at 5 years were 36.0% (range, 20.7%-59.3%) for male and 39.6% (range, 21.4%-54.8%) for female patients. Ahmedabad urban had the highest 5-year ASRS at 58.4% (95% CI, 56.3%-60.4%) for both sexes, while Manipur had the lowest rates at 20.9% (95% CI, 14.9%-27.6%). Survival rates differed significantly between rural areas (34.1%; 95% CI, 31.4%-36.9%) and urban areas (48.5%; 95% CI, 47.4%-49.7%). Pooled data from Kollam and Thiruvananthapuram revealed that individuals 65 years or older (HR, 1.76; 95% CI, 1.44-2.14) and those with distant metastasis (HR, 3.95; 95% CI, 2.78-5.60) had a significantly higher risk of death.

**Conclusions and Relevance:**

In this cohort study from India, significant survival disparities were observed among patients with OC based on demographic factors and clinical characteristics. Survival rates were lower in rural areas compared with urban regions, underscoring the inequalities in quality of care and services and emphasizing the need to improve OC survival rates in India.

## Key Points

**Question:**

What are the 5-year survival rates for patients with oral cancer (OC) diagnosed in India between 2012 and 2015, stratified by patient age, sex, stage of disease, place of residence, and histologic finding?

**Findings:**

This cohort study of 14 059 patients from 10 population-based cancer registries found an overall 5-year survival rate of 37.2%, highlighting poor outcomes and significant disparities for patients with OC in India. Localized OC was associated with better survival outcomes, while distant metastasis was associated with a 4-fold higher risk of death.

**Meaning:**

These findings suggest that promoting early detection of OC in high-risk groups, timely access to diagnostic tests, and complete treatment delivery, especially in regions with low survival, may improve OC survival rates in India.

## Teaser

This cohort study describes the 5-year survival rates among patients with oral cancer in India and assesses the association of survival with determinants including patient age, sex, and place of residence; histologic type; and the clinical extent of the disease.

## Introduction

Oral cancer (OC) encompasses various cancer types that originate in specific subsites of the oral cavity, such as the lip, tongue, gingivae, floor of the mouth, palate, buccal mucosa, and oral commissures.^1^ Globally, OC was the 16th most common cancer, with an estimated incidence of 389 846 cases in 2022.^2^ In the same year, approximately 177 258 new cases of OC were reported in Southeast Asia, making it the fourth leading cancer type in the region. Strikingly, India bears a high burden of 143 759 cases, accounting for over one-third of global OC cases and more than three-fourths of cases in Southeast Asia.^2,3^ Tobacco use (smoking or smokeless), alcohol consumption, and human papillomavirus infection are the major risk factors for OC.^1^ Data on projected cancer incidence indicate an upward trajectory for OC in India.^4^ Despite this, there is a lack of comprehensive population-based studies on OC survival, reflecting gaps in cancer control. Existing research was limited and showed lower survival rates in India.^5,6,7,8,9^ The United Nations aims to reduce premature deaths from noncommunicable diseases, including cancer, by one-third by 2030 (Sustainable Development Goal 3.4).^10^ India had initiated programs to achieve this goal, but participation in OC screenings, a key secondary prevention measure, remains low.^11^ The Global Strategy and Action Plan on Oral Health aims to advance research through population-based epidemiological studies to estimate, prevent, and manage oral diseases and conditions, including cancer.^12^

The population-based cancer registry (PBCR) serves as the cornerstone for reliable data on cancer incidence, mortality, and survival within defined populations. Population-based cancer survival stands as a pivotal indicator for health systems performance in cancer management and is increasingly used to assess the effectiveness of existing cancer control programs.^13^ The Indian Council of Medical Research–National Centre for Disease Informatics and Research recently published population-based cancer survival estimates for cervical and breast cancer from different geographic areas,^14,15^ highlighting disparities in survival rates across various regions within India.

A global study focusing on cancer survival (SURVCAN-3)^16^ revealed a significant variation in 3-year net survival rates for OC. In India, these rates ranged from 36.2% in Barshi to 60.9% in Thiruvananthapuram.^16^ However, this analysis was limited to 4 PBCRs in India. Understanding the heterogeneity in survival across geographic regions is necessary to delineate areas of high priority for interventions. The present study has been undertaken to address this gap and seeks to offer comprehensive survival insights across a broader range of population groups in India.

This report describes the 5-year survival rates among patients with OC diagnosed between January 1, 2012, and December 31, 2015, using data collected from 10 PBCRs under the National Cancer Registry Programme (NCRP). Furthermore, we assessed the association of survival rates with key determinants, including patient age, sex, and place of residence; histologic type; and the clinical extent of the disease.

## Methods

The NCRP was launched in 1981 with the objective of systematically collecting data on cancer through PBCRs and hospital-based cancer registries located across different regions of India. This analysis focused on primary OC cases as defined by *International Classification of Diseases for Oncology, Third Revision* (C01-C06) involving the base of the tongue (C01), other and unspecified parts of the tongue (C02), gums (C03), floor of the mouth (C04), palate (C05), and other and unspecified parts of the mouth (C06) with behavior code 3 (malignant), and *International Statistical Classification of Diseases, Tenth Revision* (*ICD-10*).^17^ Patients 1 year and older diagnosed between 2012 and 2015 with at least 70% follow-up by June 30, 2021, were included. The 10 eligible PBCRs cover 2.9% of India’s population, located in Kollam, Thiruvananthapuram, Mumbai, Wardha, Ahmedabad urban, Kamrup urban, Manipur, Mizoram, Sikkim, and Tripura. Ethical approval was obtained from the Institutional Ethics Committee of the Indian Council of Medical Research–National Centre for Disease Informatics and Research. Waiver of consent was obtained as the study used anonymized registry data. This study adhered to the Strengthening the Reporting of Observational Studies in Epidemiology (STROBE) guidelines for observational studies.

### Data Collection

Data collection in each registry is mainly through active methods in which data on all new cancer cases are systematically captured by trained registry staff from various sources, including hospitals, diagnostic laboratories, vital registration records, telephone calls, and house or field visits.^14,15,18^ Standardized forms ensured data consistency. However, mortality data completeness can be challenging in cancer registries due to incomplete or inaccurately certified causes of death. To address this, our study actively followed up the patients beyond routine PBCR procedures with annual follow-ups to update vital status using a multistep approach: first, linking incidence data (including patient identifiers, *ICD-10* codes, address, etc) with all-cause mortality data. Second, for unlinked cases, meticulous efforts were undertaken to locate patients through hospital records, telephone calls, field visits, and public database searches. A detailed explanation of this process is provided in eFigure 1 in Supplement 1.

To ensure data quality for survival analysis, the study assessed several indices from the PBCR data. This included the proportion of cases with microscopical verification, cases with death certificate only, and cases with missing or unknown follow-up.^19^ We also implemented quality control checks to identify and address inconsistencies, duplicates, incompatible data entries, and date errors by collaborating with the PBCRs.

### Statistical Analysis

Data were analyzed between March 15 and August 20, 2024. Survival time was measured from the date of first diagnosis to either the date of death or the date of censoring (the last known date the patient was alive). Observed survival was estimated using actuarial methods, with patients lost to follow-up considered as censored.^20^ Relative survival was determined by comparing patient survival with the expected survival of a similar group from the general population using India’s life tables, based on the Ederer-II method.^21,22^ Median survival (range; in percentages) was calculated as comparators and presented.^16,19^ Age-standardized relative survival (ASRS) was also computed, using the age of patients and the NCRP age distribution data for patients with OC from 2012 to 2016.^20,23^ Survival analyses were further stratified by age, residence, histologic type, and clinical stage at diagnosis. Log-rank testing was used to compare survival functions across these different groups.^24^ A multivariable Cox proportional hazards model was analyzed to estimate the hazard ratio (HR) and 95% CI, adjusted for sex, age, residence, histologic type, and clinical stage at diagnosis. The Cox proportional hazards regression assumption was tested using the Schoenfeld residuals test. As a sensitivity analysis, the Cox proportional hazards model was presented, including variables that violated the proportional hazards assumption.

As per the 2011 census, the taluks or subdistricts within each PBCR were classified into 3 categories: rural (rural population ≥80%), moderately rural (rural population 50%-79%), and urban (rural population <50%).^25^ The clinical extent of the disease and cancer stage was classified into localized, regional, distant metastasis, and unknown.^26^ The age-adjusted incidence rate per 100 000 was calculated using the world standard population.^27^ Additionally, Pearson correlation analysis explored the association between substance use (tobacco or alcohol) prevalence from the National Family Health Survey (NFHS) from 2019 to 2021 and ASRS in the study population.^11^ Stata software, version 14 (StataCorp LLC) with “ltable,” “strs,” and “stcox” commands facilitated the survival analysis. Two-sided *P* < .05 indicated statistical significance.

## Results

A total of 14 059 patients were diagnosed with OC with a median age at diagnosis of 55 (IQR, 45-65) years, including both male (10 380 [73.8%]) and female (3679 [26.2%]) patients. Most of the cases (10 313 [73.4%]) were from Ahmedabad urban, Mumbai, and Thiruvananthapuram. Ahmedabad urban exhibited the highest age-adjusted incidence rate of 30.0 per 100 000 among male patients. Our findings highlighted a high incidence of OC in male patients, with squamous cell carcinoma (SCC) being the most common histopathological type (12 487 [88.8%]). Notably, 13 167 cases (93.7%) were microscopically confirmed, ensuring data quality for survival analysis. However, cases with confirmation by death certificate only (227 [1.6%]) and unknown follow-up (1998 [14.2%]) were excluded from survival analysis (Table).

**Table.** Estimated Population, AAIR, and Data Quality Indices of Oral Cancer From 2012 to 2015

PBCR	Total population	Residence, %^a^		Total No. of cases	Median age, y	AAIR, %^b^		Histologic type, No. (%)		MV, No. (%)	Excluded cases, No. (%)		
		Urban	Rural			Male	Female	SCC	Non-SCC		DCO	Follow-up unknown	Total
Kollam	10 599 914	45.0	55.0	1228	62	11.9	6.0	1047 (85.3)	181 (14.7)	1108 (90.2)	0	9 (0.7)	9 (0.7)
Thiruvananthapuram	13 283 160	53.7	46.3	1594	61	13.3	5.1	1358 (85.2)	236 (14.8)	1484 (93.1)	0	174 (10.9)	174 (10.9)
Mumbai^c^	37 663 452	100	0	3983	55	14.7	6.0	3432 (86.2)	551 (13.8)	3648 (91.6)	170 (4.3)	876 (22.0)	1046 (26.3)
Wardha	5 278 170	32.5	67.5	530	54	13.2	5.4	484 (91.3)	46 (8.7)	498 (94.0)	29 (5.5)	50 (9.4)	79 (14.9)
Ahmedabad urban	24 521 403	100	0	4736	50	30.0	7.0	4407 (93.1)	329 (6.9)	4516 (95.4)	5 (0.1)	689 (14.5)	694 (14.7)
Kamrup urban	5 085 160	100	0	678	60	21.4	9.8	588 (86.7)	90 (13.3)	612 (90.3)	20 (2.9)	52 (7.7)	72 (10.6)
Manipur	12 358 605	29.2	70.8	199	59	3.0	1.4	160 (80.4)	39 (19.6)	193 (97.0)	0	18 (9.0)	18 (9.0)
Mizoram	4 660 773	52.1	47.9	173	54	7.0	3.3	159 (91.9)	14 (8.1)	169 (97.7)	0	17 (9.8)	17 (9.8)
Sikkim	2 527 949	25.2	74.8	97	55	5.5	3.6	78 (80.4)	19 (19.6)	93 (95.9)	3 (3.1)	21 (21.6)	24 (24.7)
Tripura	15 285 138	26.2	73.8	841	57	9.0	4.5	774 (92.0)	67 (8.0)	830 (98.7)	0	92 (10.9)	92 (10.9)
Total	131 263 724	NA	NA	14 059	55	NA	NA	12 487 (88.8)	1572 (11.2)	13 167 (93.7)	227 (1.6)	1998 (14.2)	2225 (15.8)

Abbreviations: AAIR, age-adjusted incidence rate; DCO, death certificate–only verification; MV, microscopic verification; NA, not applicable; PBCR, population-based cancer registry; SCC, squamous cell carcinoma.

^a^ As per the 2011 census.

^b^ From the National Cancer Registry Programme Report, 2020.

^c^ Year of diagnosis, 2012 to 2014.

**Figure 1.**
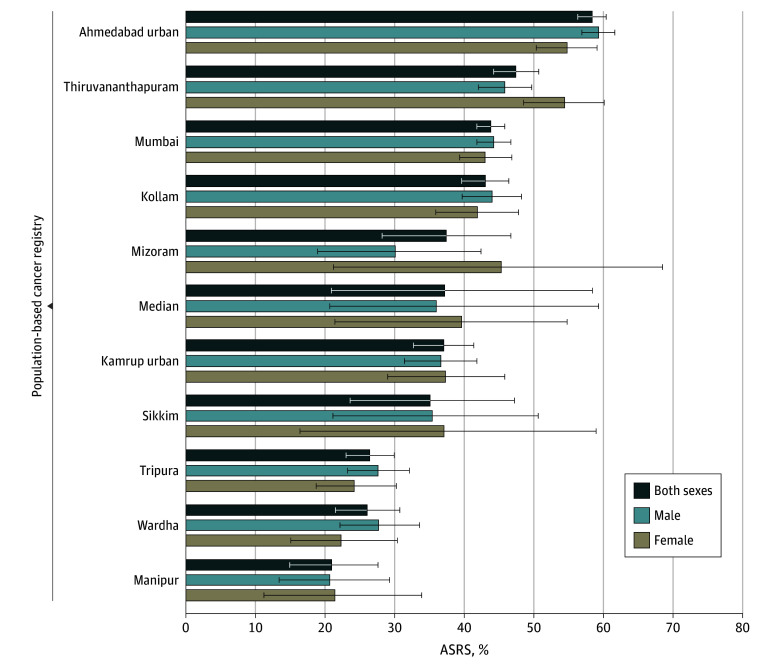
Five-Year Age-Standardized Relative Survival (ASRS) for Oral Cancer by Sex Across 10 Population-Based Cancer Registries Data are from January 1, 2012, to December 31, 2015, with follow-up to June 30, 2021. Error bars for the individual registries indicate 95% CIs; error bars for the median values indicate ranges.

Figure 1 depicts the 5-year ASRS rates across the PBCRs. The overall pooled ASRS was 37.2% (range, 20.9%-58.4%). Ahmedabad urban exhibited the highest ASRS for both sexes at the end of 5 years (58.4% [95% CI, 56.3%-60.4%] for both sexes; 59.3% [95% CI, 56.9%-61.6%] for male; 54.8% [95% CI, 50.3%-59.1%] for female). Female patients had a higher overall median 5-year ASRS (39.6%; range, 21.4%-54.8%) compared with male patients (36.0%; range, 20.7%-59.3%). In Manipur, less than a quarter of patients with OC survived for 5 years (20.9% [95% CI, 14.9%-27.6%] for both sexes; 20.7% [95% CI, 13.4%-29.3%] for male and 21.4% [95% CI, 11.2%-33.9%] for female). Female survival rates were substantially higher than male survival rates in Thiruvananthapuram and Mizoram registries.

**Figure 2.**
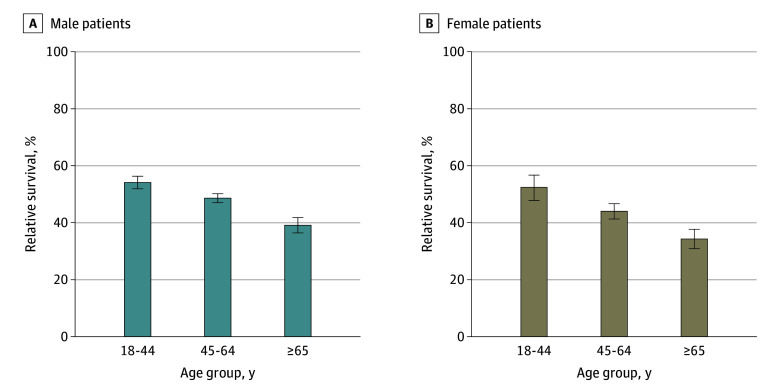
Five-Year Relative Survival by Age Group for Oral Cancer Data are from January 1, 2012, to December 31, 2015, with follow-up to June 30, 2021. Data were pooled for all 10 population-based cancer registries. Registry-wise relative survival is available in eFigure 2 in Supplement 1. Error bars indicate 95% CIs. Differences between age groups were statistically significant (*P* < .05).

Figure 2 depicts the 5-year rates of relative survival by age groups (18-44, 45-64, and ≥65 years) for male and female patients across the PBCRs. Pooled analysis revealed a consistent decline in survival with advancing age for both sexes (for male patients, from 54.1% [95% CI, 51.9%-56.3%] at 18-44 years to 48.6% [95% CI, 47.0%-50.2%] at 45-64 years and 39.1% [95% CI, 36.4%-41.8%] at ≥65 years; for female patients, from 52.4% [95% CI, 47.8%-56.7%] at 18-44 years to 44.0% [95% CI, 41.3%-46.7%] at 45-64 years and 34.3% [95% CI, 30.9%-37.7%] at ≥65 years). Statistically significant differences in survival between the age groups were noted for males in all PBCRs, except for Kamrup urban and Mizoram. Ahmedabad urban recorded the highest survival rates in all age groups for male patients (63.5% [95% CI, 60.6%-66.4%] at 18-44 years, 57.5% [95% CI, 54.9%-60.0%] at 45-64 years, and 59.3% [95% CI, 53.0%-65.4%] at ≥65 years). Conversely, Wardha had the lowest survival rate among male patients aged 18 to 44 years (27.6% [95% CI, 19.0%-36.9%]). For female patients, Thiruvananthapuram reported the highest survival (77.2% [95% CI, 55.3%-89.7%]) among the youngest group (18-44 years). Among female patients 65 years and older, Ahmedabad urban showed a peak survival rate of 52.0% (95% CI, 41.2%-62.6%). In the Northeastern registries, no significant differences in survival between age categories were observed, except in Kamrup urban (55.9% [95% CI, 35.3%-72.4%] for 18-44 years vs 29.8% [95% CI, 17.0%-44.9%] for ≥65 years) (eFigure 2 in Supplement 1).

**Figure 3.**
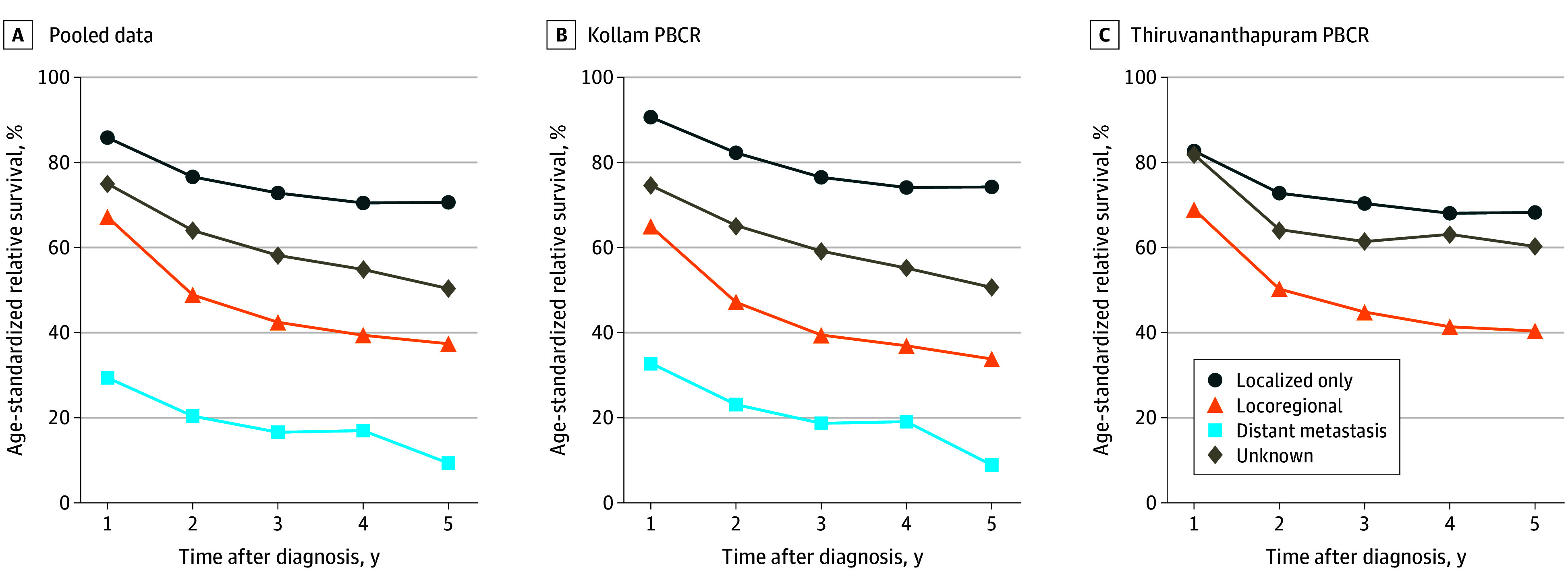
Age Standardized Relative Survival by Clinical Extent of Disease for Oral Cancer Across the Population-Based Cancer Registries (PBCRs) Data are from January 1, 2012, to December 31, 2015, with follow-up to June 30, 2021. Pooled data are from the Kollam and Thiruvananthapuram PBCRs; distance metastasis not plotted for Thiruvananthapuram due to small number of cases. Differences between the clinical extent of disease in pooled and specific PBCRs were statistically significant (*P* < .05).

Figure 3 shows ASRS for patients with OC by clinical stage at diagnosis. Data from Kollam and Thiruvananthapuram were selected for analysis due to their quality and completeness. The combined 5-year ASRS for both PBCRs were 70.6% (95% CI, 65.8%-75.1%) for localized, 37.4% (95% CI, 34.7%-40.1%) for locoregional, and 9.3% (95% CI, 3.9%-17.8%) for distant metastasis. Kollam reported the highest survival rate for localized survival at 74.2% (95% CI, 67.0%-80.6%), while Thiruvananthapuram showed a slightly better survival rate for locoregional stage at 40.4% (95% CI, 36.7%-44.1%) compared with Kollam.

**Figure 4.**
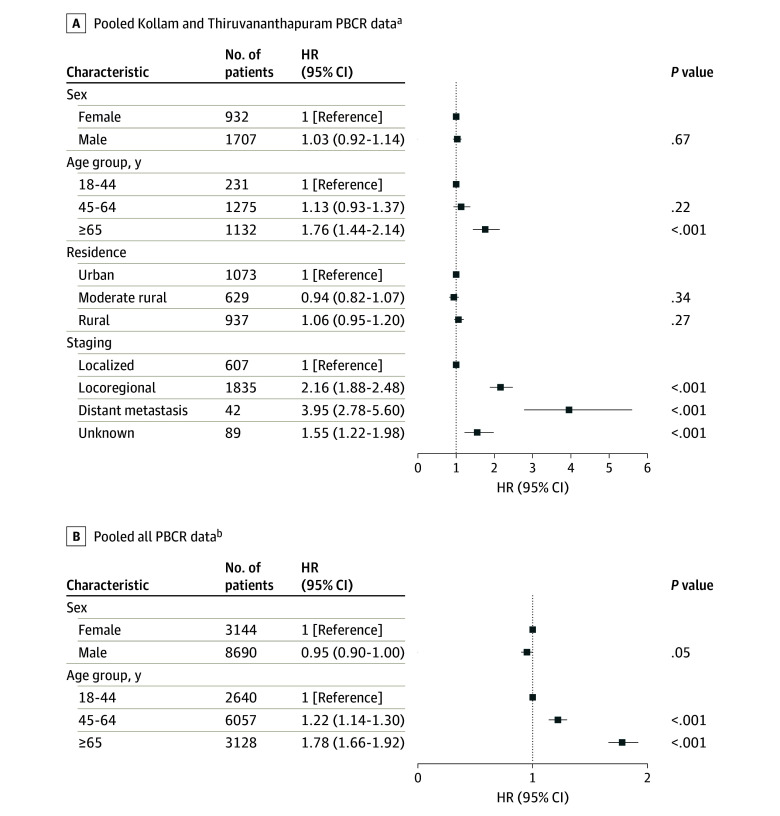
Forest Plots Depicting the Multivariable Cox Proportional Hazards Model for Oral Cancer A, Includes 1602 events; global log-rank *P* < 2 × 10^−16^ (Akaike information criterion, 22 152.9; concordance index, 0.62). B, Includes 6755 events; global log-rank *P* < 2 × 10^−16^ (Akaike information criterion, 106179.7; concordance index, 0.56). PBCR indicates population-based cancer registry. ^a^Adjusted for sex, age group, residence, and clinical extent of disease before treatment (stratified by histologic type). ^b^Adjusted for sex and age group (stratified by histologic type and residence).

The multivariable analysis of pooled data from Kollam and Thiruvananthapuram indicated that individuals 65 years or older (HR, 1.76; 95% CI, 1.44-2.14) and those with distant metastasis (HR, 3.95; 95% CI, 2.78-5.60) had a significantly higher risk of death. The pooled analysis from 10 PBCRs also revealed a significantly higher risk of death for individuals 65 years or older (HR, 1.78; 95% CI, 1.66-1.92) (Figure 4). Sensitivity analysis indicated a significantly higher risk of death for non-SCC cases (HR, 1.45; 95% CI, 1.34-1.57) and individuals from rural areas (HR, 1.30; 95% CI, 1.22-1.39) (eFigure 3 in Supplement 1).

eTable 1 in Supplement 1 details the follow-up patterns and vital status of patients included in the survival analysis (2012-2015). After excluding 2225 patients (15.8%) with unknown follow-up or verification by death certificate only, 11 834 cases were eligible for analysis, and 11 174 (94.4%) had complete 5-year follow-up. All registries maintained follow-up rates exceeding 85%, with Manipur completing 100%. Among the 11 834 patients, 4420 (37.4%) were alive 5 years after diagnosis. Manipur documented the highest proportion of deaths (150 of 181 [82.9%]) within 5 years.

The median observed survival rates for male and female patients at 5 years were 31.2% (range, 15.3%-55.5%) and 32.1% (range, 21.1%-51.8%), respectively. Ahmedabad urban exhibited the highest survival rates at all time points, with 55.5% (95% CI, 53.8%-57.2%) for male and 51.8% (95% CI, 48.1%-55.4%) for female patients at 5 years. Conversely, Manipur reported the lowest 5-year survival rates for both sexes (15.3% [95% CI, 9.6%-22.2%] for male and 21.1% [95% CI, 11.6%-32.4%] for female patients) (eTable 2 in Supplement 1).

eFigure 4 in Supplement 1 presents OC survival rates by place of residence. Pooled analysis revealed a statistically significant ASRS of 34.1% (95% CI, 31.4%-36.9%) in rural areas, 39.7% (95% CI, 36.3%-43.1%) in moderately rural areas, and 48.5% (95% CI, 47.4%-49.7%) in urban areas. Mizoram also showed a statistically significant difference in 5-year survival based on residence (18.8% [95% CI, 3.8%-43.8%] in moderately rural and 35.9% [95% CI, 24.9%-47.2%] in urban areas). Additionally, rural regions of Manipur reported a poor survival rate of 8.5% (95% CI, 0.03%-49.0%); Tripura, 21.3% (95% CI, 16.7%-26.4%); and Wardha, 21.4% (95% CI, 14.4%-29.5%).

The 5-year ASRS for SCC was significantly higher than that for non-SCC in Kollam, Thiruvananthapuram, Mumbai, and Ahmedabad urban. A pooled observed survival rate of 47.1% (95% CI, 46.0%-48.2%) was noted for SCC, while non-SCC had a lower rate of 36.5% (95% CI, 33.2%-39.8%). Non-SCC demonstrated higher survival rates in the Northeastern registries, including Kamrup urban (42.7% [95% CI, 28.4%-56.8%] vs 35.5% [95% CI, 30.9%-40.2%]), Manipur (30.7% [95% CI, 16.1%-47.0%] vs 19.6% [95% CI, 12.9%-27.4%]), and Tripura (27.7% [95% CI, 15.5%-41.7%] vs 25.9% [95% CI, 22.4%-29.6%]) (eFigure 5 in Supplement 1). eFigure 6 in Supplement 1 shows the ASRS for tongue and mouth cancer, highlighting similar median 5-year survival rates for tongue (40.5% [range, 24.9%-55.0%]) and mouth (39.3% [range, 18.7%-60.4%]) cancer.

The predominant methods used to track patient vital status were hospital visits in Thiruvananthapuram (495 of 1594 [31.1%]), telephone calls in Manipur (96 of 199 [48.2%]), and home visits in Tripura (289 of 841 [34.4%]) (eTable 3 in Supplement 1). eFigure 7 in Supplement 1 illustrates the correlation between tobacco and alcohol consumption and 5-year ASRS. In male patients, there was a strong negative correlation between substance use (tobacco use, *r* = −0.74 [*P* = .01]; alcohol use, *r* = −0.83 [*P* = .003]) and OC survival. In contrast, female patients showed a moderate negative correlation between tobacco use (*r* = −0.43; *P* = .21) and survival, and a weak negative correlation between alcohol consumption (*r* = −0.20; *P* = .58) and OC survival. eTable 4 in Supplement 1 summarizes 5-year OC survival rates from previous PBCR studies in India,^6,7,8,9,16,28,29,30,31^ highlighting the differences in survival rates across different time periods and geographic regions.

## Discussion

This study used data from 10 PBCRs, encompassing 14 059 patients diagnosed with OC, including both male (73.8%) and female (26.2%) patients. Most of the cases (73.4%) were from Ahmedabad urban, Mumbai, and Thiruvananthapuram. Among those eligible for follow-up analysis, 94.4% of patients were followed up for the entire 5-year study period. The PBCR data were processed as per the standards of International Agency for Research on Cancer and have been published in the recent *Cancer Incidence in Five Continents* volumes, ensuring their quality and completeness.^32^ The study revealed that approximately one-third of individuals diagnosed with OC in India survive for at least 5 years, though survival rates vary significantly across the country. Also, the 5-year ASRS for female patients (39.6%) was slightly higher compared with that for male patients (36.0%). Patients diagnosed with localized OC exhibited significantly better survival outcomes, while those with distant metastasis had a 4-fold higher risk of death. This study aligned with the World Health Organization Global Strategy and Action Plan on Oral Health by contributing to the monitoring of oral health status and addressing health inequalities.^12^

The study found a median survival rate of 37.2% (range, 20.9%-58.4%) for patients with OC, closely comparable to ASRS for OC in India (37.0%; range, 26.1%-45.3%) reported in SURVCAN-2 but lower than the median survival (41.6%; range, 26.5%-54.6%) reported in SURVCAN-3.^16,19^ This decrease in survival rates may be attributed to the poorer outcomes in Wardha and Northeastern registries included in our study, whereas SURVCAN-3 estimates were based on data from 4 PBCRs of Dindigul, Barshi, Kollam, and Thiruvananthapuram. India exhibits a low survival rate for OC compared with other Asian regions, such as the Republic of Korea (65.4%; range, 63.8%-66.9%) and Israel (68.9%; range, 64.6%-72.9%).^16^ In high-income countries such as the United Kingdom and US, survival rates exceed 60%.^33,34^ Significant variations in survival rates were observed across the PBCRs, with Ahmedabad urban reporting a rate of 58.4%, while Manipur had a survival rate of 20.9%. The survival disparities across PBCRs within India may reflect the differences in availability and accessibility of cancer care in these regions.^16^

Pooled data from our study indicated that urban residents had significantly better survival outcomes than rural residents, which aligns with a South Indian study that found higher cancer mortality rates for OC among rural female patients compared with their urban counterparts.^35^ Studies evaluating the role of rurality in cancer survival have noted that rural residence may serve as a proxy for a set of factors influencing access to care.^36^ People living in rural areas were more likely to be diagnosed at an advanced stage of disease compared with those in urban areas due to limited access to diagnostic services, including missed diagnoses or misdiagnoses from poorly equipped health centers.^37,38^ A study from Mizoram^39^ indicated that cancer treatment facilities were primarily concentrated in urban areas, such as Aizawl. Findings suggested that the limited access combined with the lower socioeconomic status of the rural Mizo population had contributed to higher cancer-related mortality rates. It may also be noted that behavioral risk factors for OC incidence (tobacco and alcohol use) are more prevalent in the rural than in the urban population.^40,41^ Research showed that tobacco smoking and alcohol consumption were associated with poorer survival rates in patients with oral cancer.^42,43^ Moreover, our study identified negative correlations between tobacco and/or alcohol consumption and 5-year survival rates among male patients with OC, indicating that higher substance use is associated with poorer outcomes.

The most commonly reported histologic type of OC was SCC.^44^ In our study, 88.8% of OC cases were identified as SCC, which exhibited better survival rates than other subtypes. Furthermore, the study results imply that stage of OC and age at diagnosis are associated with survival, consistent with previously established findings.^19^ Younger groups among both sexes demonstrated better survival outcomes across most registries. A meta-analysis pointed out significantly better overall survival in younger patients compared with older adults.^45^ Patients with localized disease showed significantly higher 5-year survival rates compared with those with distant metastasis, revealing a substantial absolute difference of 60%. A comparable trend was observed in the US, though with higher survival rates. Data from 22 registries showed a decline in 5-year survival rates, falling from 87.5% for localized cancer to 37.8% for distant-stage cancer.^46^

The lower survival rates will impact public health burden and economic growth. Promoting early detection of OC in high-risk groups, along with timely diagnostic evaluations and access to comprehensive cancer treatment in regions with a high disease burden, is essential for improving outcomes in OC.^12^ Studies have shown that oral visual screening in high-risk individuals significantly reduces OC mortality.^47^ However, reports from the NFHS reveal a significant gap in screening awareness, with participation rates among men and women alarmingly below 2%.^11,48^ This limited uptake of screening services likely contributed to delayed diagnoses and poorer prognosis for OC in India.

### Limitations

This study has limitations, including the exclusion of 15.8% of cases, which may bias survival estimates, and potential underreporting in rural areas due to inadequate death registration. Strengthening mortality data systems and health care facility linkages could improve future analyses. The use of recent district-level NFHS data on substance use limits temporal associations and may introduce ecological fallacy. Some PBCRs were excluded from stage-wise analysis due to possible disease stage misclassification. No imputation techniques were applied to handle missing data. Additionally, data on social and geospatial health determinants, comorbidities, treatment, and health care access were unavailable, restricting a more comprehensive survival assessment. Future research should focus on identifying the factors driving these survival differences, with an emphasis on investigating access to health care and the impact of socioeconomic status.^19,49^

## Conclusions

In this cohort study from India, significant survival disparities were observed among patients with OC based on demographic factors (eg, age and residence) and clinical characteristics (histologic type and clinical stage). Survival rates were lower in rural areas compared with urban regions, underscoring the inequalities in quality of care and services and emphasizing the need to improve OC survival rates in India.

## Notes

Supplement 1. **eFigure 1.** Outline of the Population-Based Cancer Survival Study Under the National Cancer Registry Programme **eFigure 2.** Five-Year Relative Survival (RS) by Age Group for Oral Cancer (2012-2015) **eFigure 3.** Forest Plots Depicting the Multivariable Cox Proportional Hazard Model for Oral Cancer **eTable 1.** Number and Proportions of Oral Cancer Cases With Vital Status and Follow-Up Pattern (2012-2015) **eTable 2.** Observed Survival for Oral Cancer Across the 10 PBCRs (2012-2015) **eFigure 4.** Five-Year Age Standardized Relative Survival by Place of Residence for Oral Cancer Across the PBCRs (2012-2015) **eFigure 5.** Five-Year Age Standardized Relative Survival by Histology for Oral Cancer Across the PBCRs (2012-2015) **eFigure 6.** Age-Standardized Relative Survival for Tongue and Mouth Cancer Across the PBCRs (2012-2015) for Both Sexes **eTable 3.** Methods of Follow-Up for Patients With Oral Cancer Across the PBCRs (2012-2015) **eFigure 7.** Correlation Between Survival and Consumption of Tobacco and/or Alcohol Across the PBCRs (2012-2015) **eTable 4.** Five-Year Survival From Various Studies Conducted in India on Oral Cancer
eReferences.

Supplement 2.
Data Sharing Statement
