# Supplementary material for: Geographic Disparities in Oral Cancer Survival From 10 Population-Based Cancer Registries in India
Source: JAMA Netw Open. 2025 Apr 8;8(4):e253910. doi: 10.1001/jamanetworkopen.2025.3910 (PMC11979727; doi:10.1001/jamanetworkopen.2025.3910)
Supplement: Supplement 1. — eFigure 1. Outline of the Population-Based Cancer Survival Study Under the National Cancer Registry Programme eFigure 2. Five-Year Relative Survival (RS) by Age Group for Oral Cancer (2012-2015) eFigure 3. Forest Plots Depicting the Multivariable Cox Proportional Hazard Model for Oral Cancer eTable 1. Number and Proportions of Oral Cancer Cases With Vital Status and Follow-Up Pattern (2012-2015) eTable 2. Observed Survival for Oral Cancer Across the 10 PBCRs (2012-2015) eFigure 4. Five-Year Age Standardized Relative Survival by Place of Residence for Oral Cancer Across the PBCRs (2012-2015) eFigure 5. Five-Year Age Standardized Relative Survival by Histology for Oral Cancer Across the PBCRs (2012-2015) eFigure 6. Age-Standardized Relative Survival for Tongue and Mouth Cancer Across the PBCRs (2012-2015) for Both Sexes eTable 3. Methods of Follow-Up for Patients With Oral Cancer Across the PBCRs (2012-2015) eFigure 7. Correlation Between Survival and Consumption of Tobacco and/or Alcohol Across the PBCRs (2012-2015) eTable 4. Five-Year Survival From Various Studies Conducted in India on Oral Cancer eReferences. [file jamanetwopen-e253910-s001.pdf]

Supplementary Online Content

Sathishkumar K, Sankarapillai J, Santhappan S, et al. Geographic disparities in oral cancer survival from 10 population-based cancer registries in India. *JAMA Netw Open.* 2025;8(4):e253910. doi:10.1001/jamanetworkopen.2025.3910

eFigure 1. Outline of the Population-Based Cancer Survival Study Under the National Cancer Registry Programme

eFigure 2. Five-Year Relative Survival (RS) by Age Group for Oral Cancer (2012-2015)

eFigure 3. Forest Plots Depicting the Multivariable Cox Proportional Hazard Model for Oral Cancer

eTable 1. Number and Proportions of Oral Cancer Cases With Vital Status and Follow-Up Pattern (2012-2015)

eTable 2. Observed Survival for Oral Cancer Across the 10 PBCRs (2012-2015)

eFigure 4. Five-Year Age Standardized Relative Survival by Place of Residence for Oral Cancer Across the PBCRs (2012-2015)

eFigure 5. Five-Year Age Standardized Relative Survival by Histology for Oral Cancer Across the PBCRs (2012-2015)

eFigure 6. Age-Standardized Relative Survival for Tongue and Mouth Cancer Across the PBCRs (2012-2015) for Both Sexes

eTable 3. Methods of Follow-Up for Patients With Oral Cancer Across the PBCRs (2012-2015)

eFigure 7. Correlation Between Survival and Consumption of Tobacco and/or Alcohol Across the PBCRs (2012-2015)

eTable 4. Five-Year Survival From Various Studies Conducted in India on Oral Cancer

eReferences.

This supplementary material has been provided by the authors to give readers additional information about their work.

eFigure 1. Outline of the Population-Based Cancer Survival Study Under the National Cancer Registry Programme

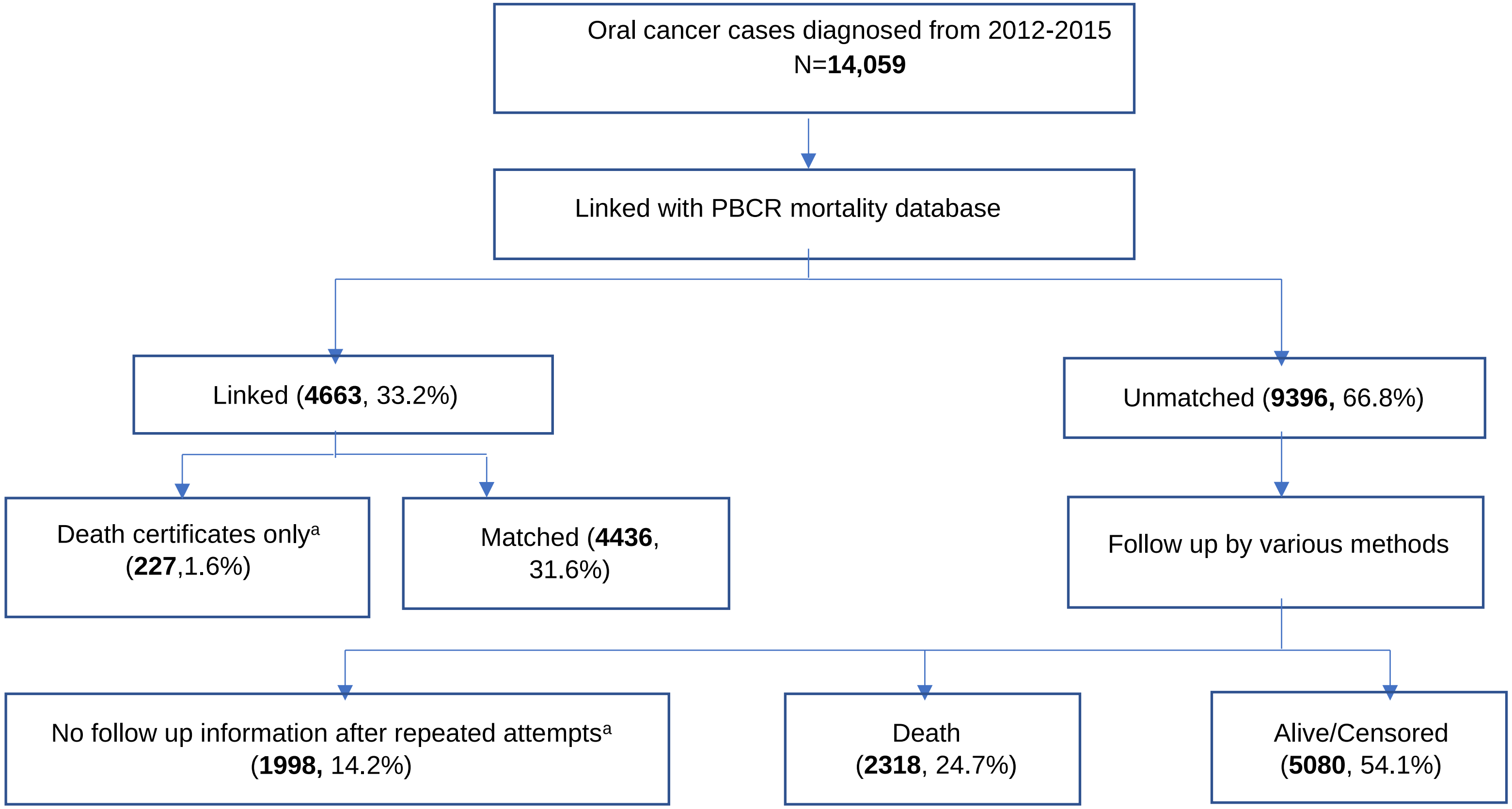

[a] Excluded from survival analysis. PBCR indicates population-based cancer registry

© 2025 Sathishkumar K et al. *JAMA Network Open*.

eFigure 2. Five-Year Relative Survival (RS) by Age Group for Oral Cancer (2012-2015)

MALES

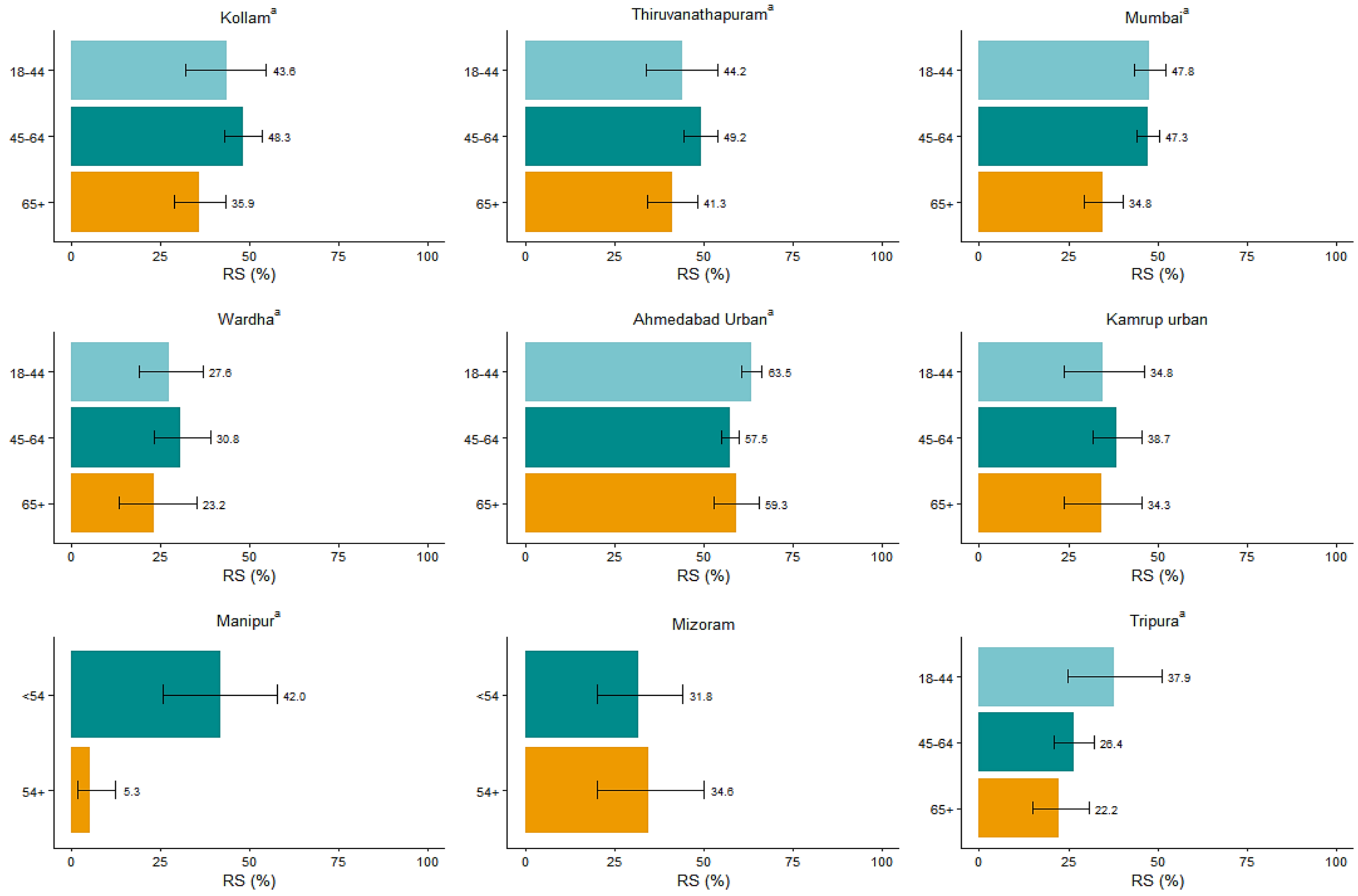

© 2025 Sathishkumar K et al. *JAMA Network Open*.

FEMALES

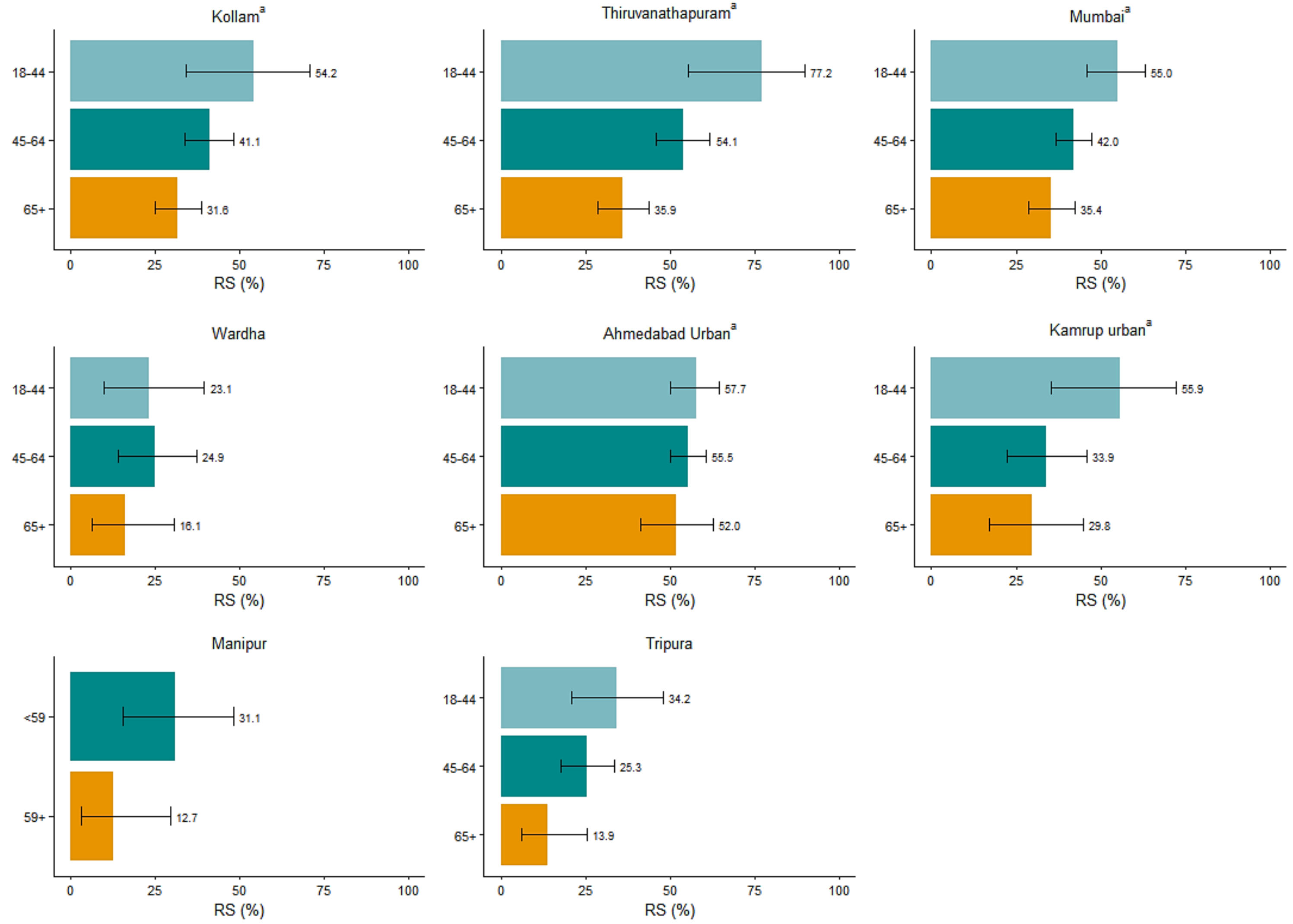

Note: Due to small numbers in Manipur (both sexes) and Mizoram (males), age categorization was done based on the median age. Sikkim (both sexes) & Mizoram (females), with fewer cases in each age category, was excluded from analysis; [a]Indicates statistically significant with p value < 0.05

© 2025 Sathishkumar K et al. *JAMA Network Open*.

eFigure 3. Forest Plots Depicting the Multivariable Cox Proportional Hazard Model for Oral Cancer

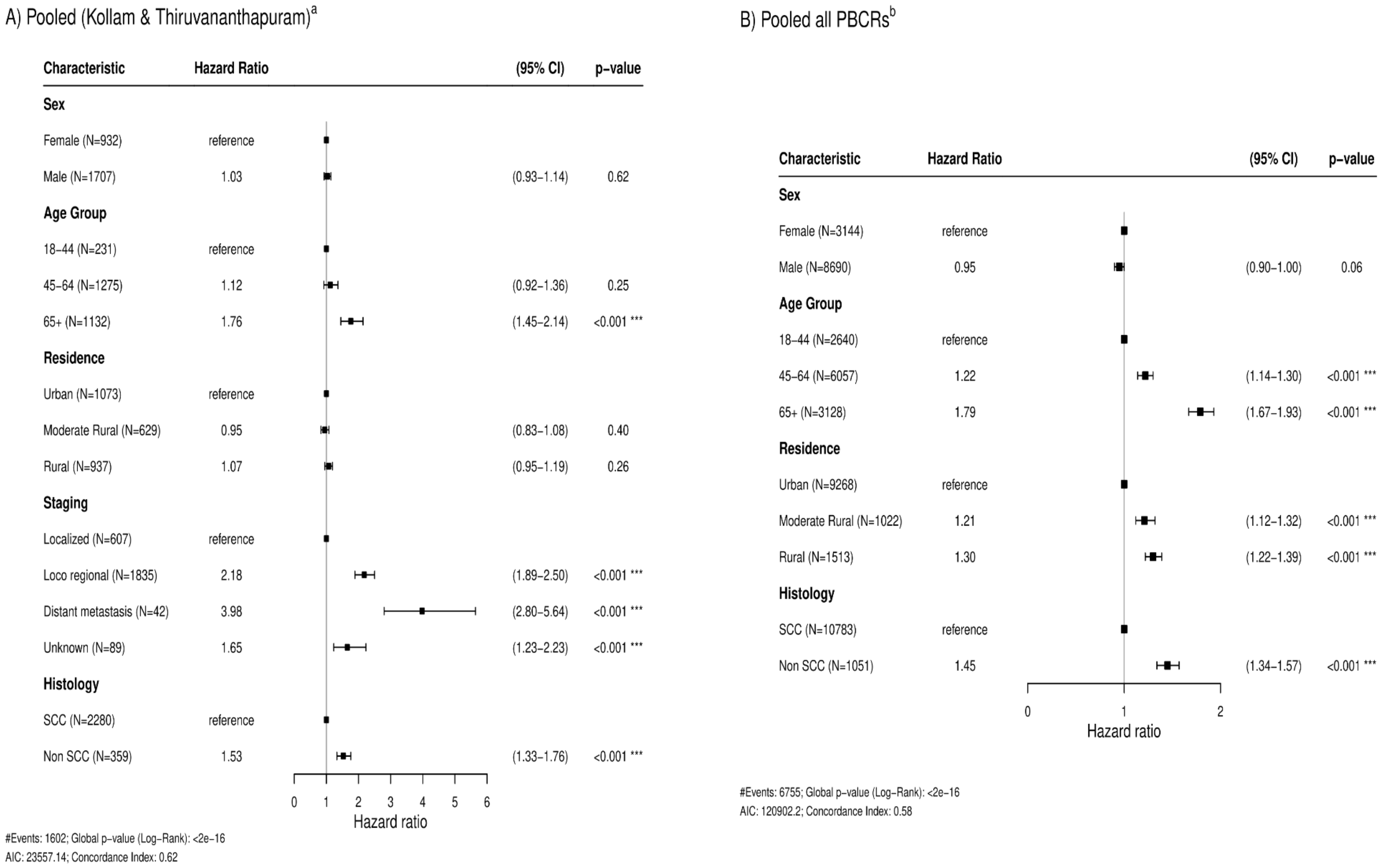

[a]Adjusted for sex, age group, residence, histology & clinical extent of disease before treatment, [b] Adjusted for sex and age group, residence & histology

© 2025 Sathishkumar K et al. *JAMA Network Open*.

eTable 1. Number and Proportions of Oral Cancer Cases With Vital Status and Follow-Up Pattern (2012-2015)

PBCR	Total Included cases	Deaths within 5 years of diagnosis		Alive 5 years from diagnosis		Complete follow-up [a] (till 5 years) %	Incomplete follow up (Lost to follow up)		
		n	%	n	%		< 1 year	1-3 years	3-5 years
Kollam	1219 (99.3)	791	64.9	423	34.7	99.6	0	0	5
Thiruvananthapuram	1420 (89.1)	811	57.1	452	31.8	88.9	62	30	65
Mumbai	2937 (73.7)	1764	60.1	994	33.8	93.9	0	16	163
Wardha	451 (85.1)	340	75.4	96	21.3	96.7	1	2	12
Ahmedabad Urban	4042 (85.3)	1781	44.1	2120	52.4	96.5	98	31	12
Kamrup Urban	606 (89.4)	394	65.0	127	21.0	86.0	9	16	60
Manipur	181 (91.0)	150	82.9	31	17.1	100.0	0	0	0
Mizoram	156 (90.2)	104	66.7	41	26.3	92.9	0	0	11
Sikkim	73 (75.3)	51	69.9	19	26.0	95.9	0	0	3
Tripura	749 (89.1)	568	75.8	117	15.6	91.5	1	2	61
**Pooled**	**11,834 (84.2)**	**6754**	**57.1**	**4420**	**37.4**	**94.4**	**171**	**97**	**392**

[a] Calculated from the total included cases

© 2025 Sathishkumar K et al. *JAMA Network Open*.

eTable 2. Observed Survival for Oral Cancer Across the 10 PBCRs (2012-2015)

PBCR	Observed Survival (95% CI)		
	1 year	3 years	5 years
**Males**			
Kollam[a]	67.5 (64.0-70.8)	44.8 (41.1-48.3)	37.8 (34.3-41.3)
Thiruvananthapuram	69.3 (66.2-72.1)	45.3 (42.0-48.5)	39.8 (36.6-42.9)
Mumbai	63.4 (61.3-65.4)	47.1 (44.9-49.2)	40.4 (38.3-42.5)
Wardha	55.1 (49.5-60.3)	29.4 (24.5-34.4)	25.5 (20.8-30.4)
Ahmedabad Urban	79.2 (77.7-80.5)	62.7 (61.0-64.3)	55.5 (53.8-57.2)
Kamrup Urban	74.6 (70.2-78.4)	42.6 (37.9-47.2)	32.4 (27.9-36.9)
Manipur[a]	52.4 (43.3-60.8)	19.4 (13.0-26.7)	15.3 (9.6-22.2)
Mizoram	52.8 (42.9-61.8)	34.0 (25.1-43.0)	29.9 (21.5-38.8)
Sikkim	45.8 (31.4-59.1)	31.3 (18.9-44.5)	29.2 (17.2-42.3)
Tripura	63.7 (59.3-67.7)	36.6 (32.3-40.8)	23.5 (19.8-27.4)
**Median (range)**	63.6 (45.8-79.2)	39.6 (19.4-62.7)	31.2 (15.3-55.5)
**Females**			
Kollam[a]	64.8 (60.4-68.9)	39.7 (35.4-44.0)	31.1 (27.0-35.2)
Thiruvananthapuram	68.1 (63.5-72.2)	45.6 (40.8-50.2)	40.2 (35.4-44.9)
Mumbai	59.9 (56.4-63.2)	44.4 (41.0-47.8)	37.3 (34.0-40.7)
Wardha	46.2 (37.4-54.4)	26.1 (18.9-33.9)	21.1 (14.5-28.6)
Ahmedabad Urban	75.7 (72.4-78.7)	58.5 (54.8-62.0)	51.8 (48.1-55.4)
Kamrup Urban	67.8 (60.0-74.4)	40.3 (32.7-47.9)	32.1 (24.8-39.7)
Manipur[a]	68.4 (54.7-78.8)	31.6 (20.1-43.7)	21.1 (11.6-32.4)
Mizoram	62.0 (47.1-73.8)	42.0 (28.3-55.1)	39.7 (26.2-52.9)
Sikkim	64.0 (42.2-79.4)	44.0 (24.5-61.9)	32.0 (15.2-50.2)
Tripura	58.1 (51.8-63.9)	31.2 (25.6-36.9)	21.9 (16.9-27.3)
**Median (range)**	64.4 (46.2-75.7)	41.2 (26.1-58.5)	32.1 (21.1-51.8)

Abbreviations: CI, confidence interval; OS, observed survival; PBCR, population-based cancer registry; [a]Indicates statistically significant with p value < 0.05

© 2025 Sathishkumar K et al. *JAMA Network Open*.

eFigure 4. Five-Year Age Standardized Relative Survival by Place of Residence for Oral Cancer Across the PBCRs (2012-2015)

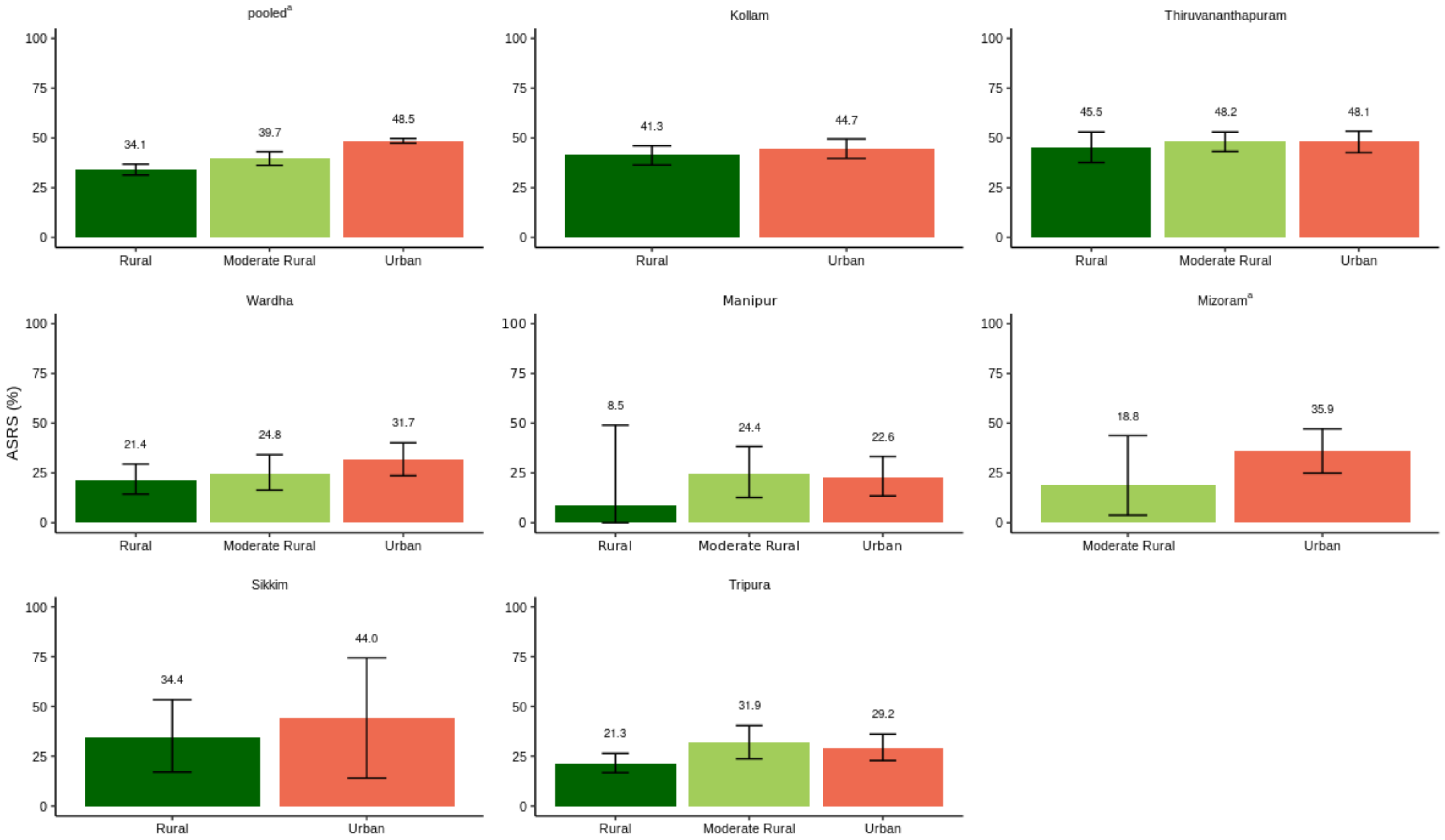

Note: Rural: Taluk with rural population proportion greater >= 80, Moderate rural: Taluk with rural population proportion between 50 to 79, Urban: Taluk with rural population proportion < 50; Ahmedabad, Mumbai and Kamrup (completely urban) not plotted separately, included in pooled analysis; [a]Indicates statistically significant with p value < 0.05; Error bars indicate 95% confidence intervals

© 2025 Sathishkumar K et al. *JAMA Network Open*.

eFigure 5. Five-Year Age Standardized Relative Survival by Histology for Oral Cancer Across the PBCRs (2012-2015)

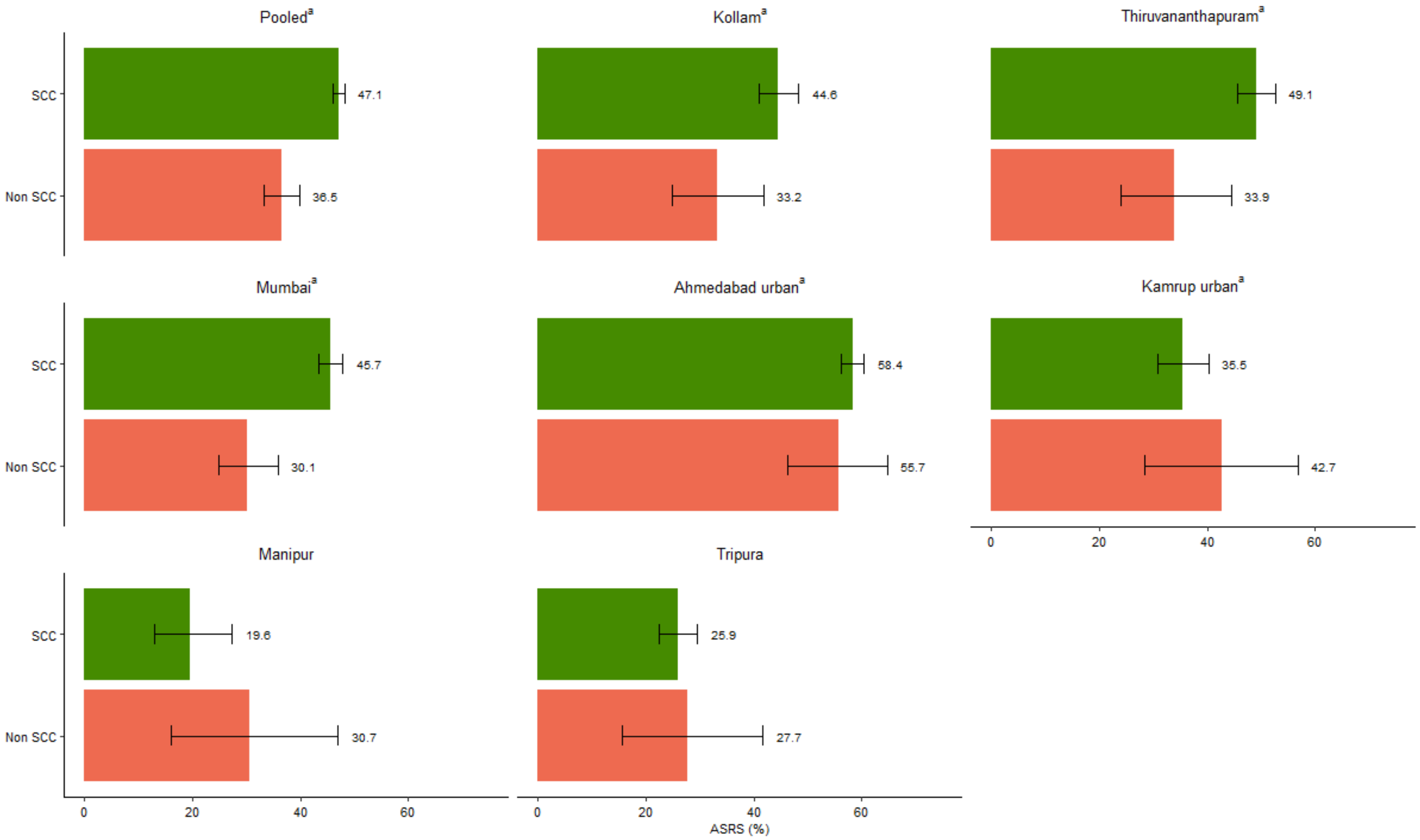

Note: SCC: Squamous cell carcinoma, Non-SCC: Non- Squamous cell carcinoma (adenocarcinoma, sarcoma, others)
Due to small numbers, Mizoram, Sikkim and Wardha not plotted but included in pooled analysis;
[a]Indicates statistically significant with p value < 0.05; Error bars indicate 95% confidence intervals

© 2025 Sathishkumar K et al. *JAMA Network Open*.

eFigure 6. Age-Standardized Relative Survival for Tongue and Mouth Cancer Across the PBCRs (2012-2015) for Both Sexes

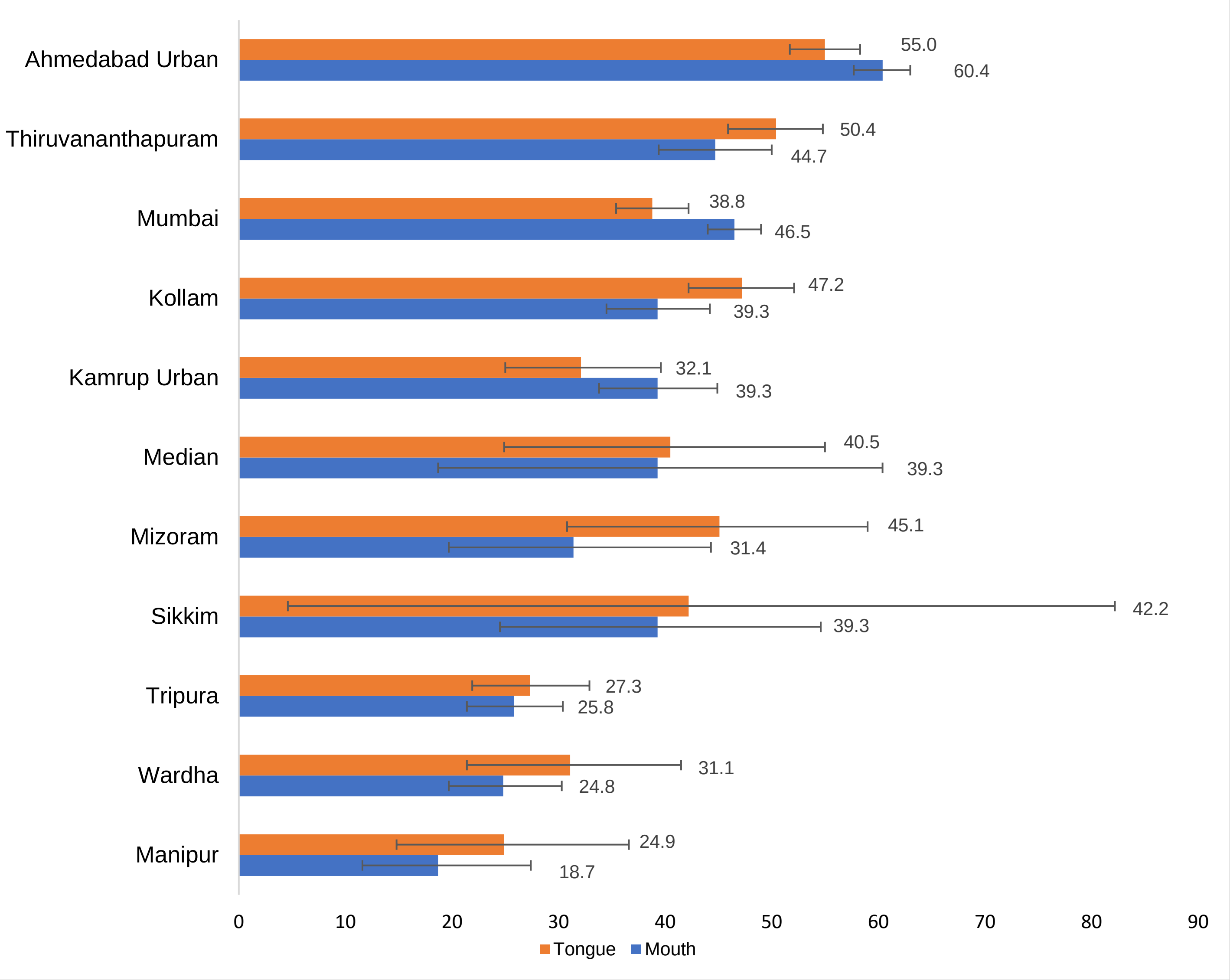

Note: Median (range); Error bars indicate 95% confidence intervals

© 2025 Sathishkumar K et al. *JAMA Network Open*.

eTable 3. Methods of Follow-Up for Patients With Oral Cancer Across the PBCRs (2012-2015)

	Hospital Visit		Post		Telephone		Home Visit		Public Database		No follow up/DCO		Others		Linked with mortality database		Total
	n	%	n	%	n	%	n	%	n	%	n	%	n	%	n	%	n
Kollam	23	1.9	1	0.1	357	29.1	15	1.2	0	0.0	9	0.7	302	24.6	521	42.4	1228
Thiruvanant hapuram	495	31.1	1	0.1	6	0.4	10	0.6	169	10.6	174	10.9	0	0.0	739	46.4	1594
Mumbai	342	8.6	1	0.03	698	17.5	153	3.8	349	8.8	1046	26.3	8	0.2	1386	34.8	3983
Wardha	13	2.5	0	0.0	77	14.5	34	6.4	1	0.2	79	14.9	2	0.4	324	61.1	530
Ahmedabad Urban	143	3.0	1	0.02	2,681	3.0	278	5.9	4	0.1	694	14.7	21	0.4	914	19.3	4736
Kamrup Urban	1	0.2	0	0.0	241	35.6	203	29.9	0	0.0	72	10.6	67	9.9	94	13.9	678
Manipur	3	1.5	0	0.0	96	48.2	64	32.2	1	0.5	18	9.0	2	1.0	15	7.5	199
Mizoram	1	0.6	0	39.9	69	0.6	9	5.2	1	0.6	17	9.8	0	0.0	76	48.7	173
Sikkim	3	3.1	0	0.0	28	28.9	8	8.3	0	0.0	24	24.7	0	0.0	34	35.1	97
Tripura	12	1.4	0	0.0	94	11.2	289	34.4	20	2.4	92	10.9	1	0.1	333	39.4	841
Total	**1,036**	**7.4**	**4**	**0.03**	**4,347**	**30.9**	**1,063**	**7.6**	**545**	**3.9**	**2225**	**15.8**	**403**	**2.9**	**4436**	**31.6**	**14059**

Note: Patients were followed up on multiple times. This table summarizes the most recent method of follow-up

© 2025 Sathishkumar K et al. *JAMA Network Open*.

eFigure 7. Correlation Between Survival and Consumption of Tobacco and/or Alcohol Across the PBCRs (2012-2015)

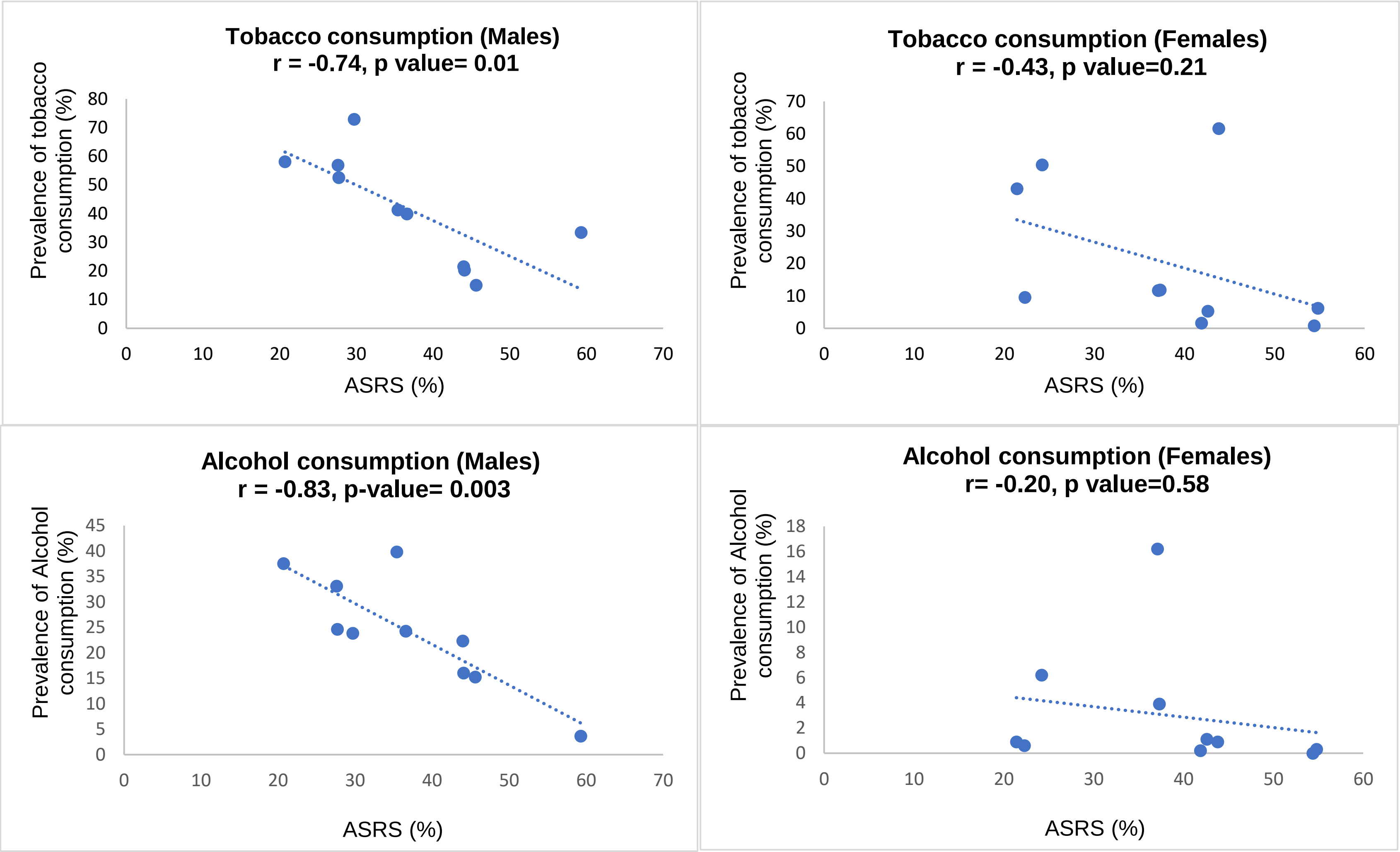

Data source: State and District Fact sheets, National Family Health Survey-5 (2019-21); ASRS, Age Standardized Relative Survival

© 2025 Sathishkumar K et al. *JAMA Network Open*.

eTable 4. Five-Year Survival From Various Studies Conducted in India on Oral Cancer

Study	Time Period	Year of Publication	PBCR	Parameter measured	ICD 10 Site code	Survival (%)
Yeole BB et al[1]	1987– 1991	2000	Mumbai	5-year ASRS	ICD-9 140<br>ICD-9 141<br>ICD-9 141<br>ICD-9 143–145	Base of tongue: 24.3<br>Anterior Tongue: 49.4<br>Mouth: 47.1
R Swaminathan et al[2]	2003-2006	2009	Dindigul	ASRS	C01−02<br>C03−06	Tongue: 37.9<br>Mouth: 41.9
R. Sankaranarayanan et al[3]	2002-2008	2010	India	ASRS	C01−02<br>C03−06	Tongue: 37 (26–45)<br>Oral cavity: 23 (12–30)
K Jayant et al[4 6]	1993-2003	2011	Barshi	ASRS	C01−02<br>C03−06	Tongue: 10.4<br>Oral cavity: 22.9
R Dikshit et al[5 7]	1991-2000	2011	Bhopal	ASRS	C01−02<br>C03−06	Tongue: 11.7<br>Oral cavity: 33.9
R Swaminathan et al[6 9]	1990-2001	2011	Chennai	ASRS	C00<br>C01−02<br>C03−06	Tongue: 23.2<br>Oral cavity: 35.6
P. Jayalekshmi et al[7]	1991-1999	2011	Karunagappally	ASRS	C01−02<br>C03−06	Tongue: 31.1<br>Oral cavity: 42.3
Yeole BB et al[8]	1992-2003	2011	Mumbai	ASRS	C00<br>C01−02<br>C03−06	Tongue: 27.8<br>Oral cavity: 35.0
SURVCAN-3[9]	2008-2011	2023	Barshi	ASRS	C01-06	28.7 (17.3-41.1)
	2008-2012		Dindigul	ASRS	C01-06	26.5 (20.4-33.0)
	2008-2011		Kollam	ASRS	C01-06	54.6 (50.6-58.3)
	2008-2011		Trivandrum	ASRS	C01-06	54.5 (47.8-60.6)

© 2025 Sathishkumar K et al. *JAMA Network Open*.

eReferences.

1. Yeole, B. B., Sankaranarayanan, R., Sunny M Sc, L., Swaminathan, R., & Parkin, D. M. (2000). Survival from head and neck cancer in Mumbai (Bombay), India. *Cancer*, *89*(2), 437–444. https://doi.org/10.1002/1097-0142(20000715)89:2<437::aid-cncr32>3.0.co;2-r
2. Swaminathan, R., Selvakumaran, R., Esmy, P. O., Sampath, P., Ferlay, J., Jissa, V., Shanta, V., Cherian, M., & Sankaranarayanan, R. (2009). Cancer pattern and survival in a rural district in South India. *Cancer epidemiology*, *33*(5), 325–331. https://doi.org/10.1016/j.canep.2009.09.008
3. Sankaranarayanan R, Swaminathan R, Brenner H, et al. Cancer survival in Africa, Asia, and Central America: a population-based study. *The Lancet Oncology*. 2010;11(2):165-173. doi:10.1016/S1470-2045(09)70335-3
4. Jayant, K., Nene, B. M., Dinshaw, K. A., Badwe, R. A., Panse, N. S., & Thorat, R. V. (2011). Cancer survival in Barshi, India, 1993-2000. *IARC scientific publications*, (162), 101–106.
5. Dikshit, R., Kanhere, S., & Surange, S. (2011). Cancer survival in Bhopal, India, 1991-1995. *IARC scientific publications*, (162), 107–113.
6. Swaminathan, R., Rama, R., Nalini, S., & Shanta, V. (2011). Cancer survival in Chennai (Madras), India, 1990-1999. *IARC scientific publications*, (162), 115–124.
7. Jayalekshmi, P., Gangadharan, P., & Sebastian, P. (2011). Cancer survival in Karunagappally, India, 1991-1997. *IARC scientific publications*, (162), 125–132.
8. Yeole, B. B., Kurkure, A. P., & Sunny, L. (2011). Cancer survival in Mumbai (Bombay), India, 1992-1999. *IARC scientific publications*, (162), 133–142.
9. Soerjomataram, I., Cabasag, C., Bardot, A., Fidler-Benaoudia, M. M., Miranda-Filho, A., Ferlay, J., Parkin, D. M., Ranganathan, R., Piñeros, M., Znaor, A., Mery, L., Joko-Fru, Y. W., Dikshit, R., Sankaranarayanan, R., Swaminathan, R., Bray, F., & SURVCAN-3 collaborators (2023). Cancer survival in Africa, central and south America, and Asia (SURVCAN-3): a population-based benchmarking study in 32 countries. *The Lancet. Oncology*, *24*(1), 22–32. https://doi.org/10.1016/S1470-2045(22)00704-5

© 2025 Sathishkumar K et al. *JAMA Network Open*.
