# Supplementary material for: Geographic Disparities in Oral Cancer Survival From 10 Population-Based Cancer Registries in India
Source: JAMA Netw Open. 2025 Apr 8;8(4):e253910. doi: 10.1001/jamanetworkopen.2025.3910 (PMC11979727; doi:10.1001/jamanetworkopen.2025.3910)
Supplement: Supplement 2. — Data Sharing Statement [file jamanetwopen-e253910-s002.pdf]

Data Sharing Statement

Sathishkumar. Geographic Disparities in Oral Cancer Survival From 10 Population-Based Cancer Registries in India. *JAMA Netw Open*. Published April 08, 2025. doi:10.1001/jamanetworkopen.2025.3910

Data
Data available: Yes
Data types: Deidentified participant data
How to access data: De-identified data collected for this study are available upon a reasonable request over email to the corresponding author
When available: With publication

Supporting Documents
Document types: None

Additional Information
Who can access the data: Researchers whose proposed use of the data has been approved
Types of analyses: Population based survival data
Mechanisms of data availability: After approval of a proposal along with a signed data access agreement
